# Building up and breaking down: co‐ordination of cell wall production and proteasome assembly

**DOI:** 10.1111/febs.70135

**Published:** 2025-05-19

**Authors:** Thomas D. Williams

**Affiliations:** ^1^ Sir William Dunn School of Pathology University of Oxford UK

**Keywords:** cell wall, cell warfare, proteasome, stress adaptation, yeast

## Abstract

Cell wall maintenance and proteome remodelling are fundamental requirements for fungal cells subjected to stress. Previous work has shown that the cell wall integrity pathway is activated upon diverse stresses to both increase the production of cell wall constituents and proteasome assembly factors through activation of Mpk1 kinase. In a recent study, Šupljika and colleagues identified a further link between the two processes. The E3 ligase adaptor Mub1 is a negative regulator of both proteasome subunit production and cell wall maintenance factors. These multifactoral links may have emerged from interspecies competition, providing a benefit to linking these diverse stress adaptation mechanisms.

## Graphical abstract

Cell wall maintenance and proteome remodelling are fundamental requirements for fungal cells subjected to stress. Previous work has shown that the cell wall integrity pathway is activated upon diverse stresses to both increase production of cell wall constituents and proteasome assembly factors through activation of Mpk1 kinase. In a recent study, Šupljika and colleagues identified a further link between the two processes. The E3 ligase adaptor Mub1 is a negative regulator of both proteasome subunit production and cell wall maintenance factors. These multifactoral links may have emerged from interspecies competition, providing a benefit to linking these diverse stress adaptation mechanisms.

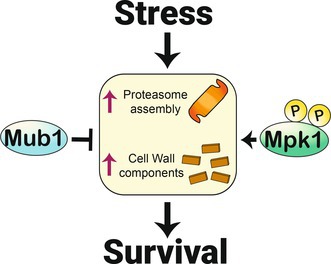

Comment on: https://doi.org/10.1111/febs.70091.

**Abbreviations**
CWI: cell wall integrity
ER: endoplasmic reticulum
TORC1: target of rapamycin complex 1

## Introduction

Proteasomes and the budding yeast cell wall are, on the face of it, quite different. One acts to destroy proteins, which have become damaged, misfolded or are otherwise not required, while the other forms a sturdy barrier around the cell. The former is required for protein homeostasis maintenance, while the latter is essential to maintain cell wall integrity (CWI) and prevent cell lysis under normal conditions. Despite these differences, protein homeostasis and CWI share one clear similarity: They are hugely expensive processes. Investment in them must be limited to what is essential, or the cell will cease to be evolutionarily competitive.

There is now a growing body of evidence linking cell wall production and cellular proteasome levels. Both are increased under similar stress conditions, with proteasome assembly dependent upon CWI signalling. In this issue of *FEBS Journal*, Šupljika and colleagues detail how an *S. cerevisiae* E3 ligase adaptor important for limiting proteasome production additionally limits production of cell wall components upon the application of cell wall stressors, tying these two processes closer together [1].

## Co‐ordination of proteasome and cell wall component production

**Fig. 1:**
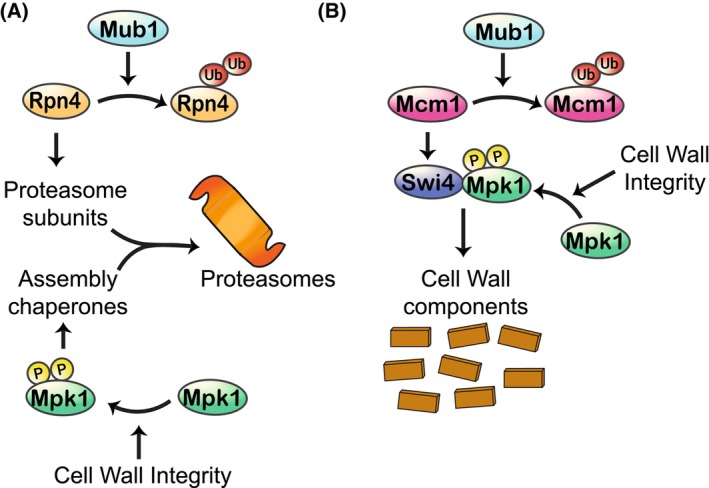
Co‐operation of Mpk1 and Mub1 in proteasome production and cell wall maintenance. (A) Roles in proteasome production. Mub1 limits the level of Rpn4, reducing the pool of proteasome subunits. Active Mpk1 is required for the translation of proteasome regulatory particle assembly chaperones. The assembly chaperones are essential to help the proteasome subunits assemble into the final proteasome. (B) Roles in cell wall maintenance. Mub1 limits the levels of the Mcm1 transcription factor. Mcm1 increases the production of the Swi4 transcription factor. Active Mpk1 binds Swi4 to activate transcription of enzymes controlling the production of cell wall components.

Under normal laboratory conditions, proteasome assembly and cell wall production are both kept relatively low. Proteasome subunit levels are regulated by the transcription factor Rpn4 [2], which is degraded by the proteasome following ubiquitination mediated by the Mub1 E3 ligase adaptor [3]. In an elegant and conserved feedback loop, during conditions requiring more protein degradation, such as endoplasmic reticulum (ER) stress and nutrient deprivation, Rpn4 is not degraded, and more proteasome components are produced. These are then assembled into proteasomes with the aid of assembly chaperones, whose production is regulated by the MAPKinase Mpk1 (Fig. 1A) [4]. Intriguingly, Mpk1 is activated via the CWI pathway, inducing its own production while also restricting its activating mechanisms: controlling both positive and negative feedback loops [3]. Correspondingly, treatment with cell wall stressors is sufficient to drive proteasome precursor production [4, 5]. Mpk1 activation is the final core step in the CWI signalling pathway and activates various transcription factors controlling the production of cell wall components, including Swi4 (Fig. 1B).

Šupljika *et al*. [1] have deepened the link between these processes. They demonstrate that in the absence of the Mub1 E3 ligase adaptor, cells are hyper resistant to cell wall stress, as they are better able to maintain cell wall mannoprotein content. This was not related to Mub1's role in proteasome regulation, rather it regulates levels of the transcription factor, Mcm1 (Fig. 1B). Mcm1 is more highly expressed in the absence of Mub1, likely driving the higher transcriptional activity of Mcm1 targets they observed. Cells bearing Mcm1 loss‐of‐function alleles are more sensitive to cell wall stressors [6], while overexpression can increase resistance [1]. Another CWI transcription factor, Swi4, is also essential for this process. Mcm1 can regulate Swi4 levels [7], likely placing it upstream of Swi4 in this system (Fig. 1B). Swi4 and activated Mpk1 can interact noncatalytically to regulate transcription of an enzyme regulating cell wall biosynthesis: *FKS2*, which encodes a stress induced 1,3‐beta‐glucan synthase [8]. Intriguingly, in the absence of Mub1 there is no excess cell wall component accumulation in unstressed cells despite elevated Mcm1 levels [1]. This may be because differences in downstream factors, such as Swi4 are relatively minor, and the minimal levels of CWI pathway activation restrict the amount of activated Mpk1 available. There are therefore several elements of cross‐regulation between proteasome production and cell wall maintenance upon stress.

Why is it desirable to co‐ordinate cell wall and proteasome production? When the cell wall is under threat, cells need to redeploy resources to fortify the wall and limit growth accordingly. The proteins which would otherwise be employed to help the cell grow are then no longer needed and may be actively harmful. Their constituent molecules can instead be utilised in keeping the cell alive by building the wall. Similarly, situations where large amounts of protein turnover are required, such as nutrient starvation or ER stress, are those where growth has to be repressed and survival prioritised. With this in mind, it is notable that the CWI pathway is activated upon several conditions which do not have a clear link to the cell wall [9], probably through the inhibition of the environmentally responsive TORC1 kinase complex. Increased cell wall production is therefore a general cytoprotective mechanism.

## An evolutionary basis for cell wall production upon stress

**Fig. 2:**
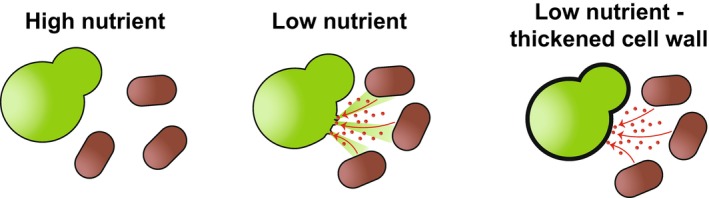
Possible role of thickened cell wall during nutrient starvation in an ecological context. In high nutrient environments, fungi and bacteria can happily live alongside each other (left). When nutrient levels drop, antagonistic relationships can occur, with the bacteria producing cell wall‐degrading compounds to obtain nutrients from fungal lysis (middle). A thickened fungal cell wall likely helps survival in this context (right).

A thickened cell wall is unlikely to be a requirement for surviving nutrient depletion *per se*. It is notable however that rapamycin treatment, which mimics nitrogen starvation, causes a rapid increase in resistance to the cell wall‐degrading enzyme cocktail Zymolyase [5]. This unexpected link may be explained by consideration of the evolutionary history of *S. cerevisiae*. *S. cerevisiae* likely evolved as part of a complex community (Fig. 2, left) on Taiwanese plants [10] before its domestication and subsequent use in food products and as a model organism. Unlike in a laboratory setting where individual nutrients can be depleted with no other perturbations, in this real‐world scenario, the onset of nutrient stress is likely the beginning of fierce competition for limited resources between the local microflora, including bacteria and other fungi. A prime way for a bacterium to obtain more nutrients is to release them into the environment by lysing the local fungi (Fig. 2, middle). Genes encoding fungal cell wall‐degrading enzymes are widespread within the bacterial community [11], with some bacteria able to utilise fungi as their sole nutrient source [12]. It is important to note that these relationships are not always antagonistic: mutually beneficial interactions are also common within bacterial–fungal interactions [13]. In the antagonistic situation of organismal warfare over limited resources, a thickened cell wall is a requirement to protect the cell from external competition, while proteasome production is paramount for making the best use of the available intracellular resources (Fig. 2, right). While this remains speculative, it is a powerful and attractive example of how evolutionary context may impact axenic laboratory cultures in unexpected ways.

## Conclusion

The work presented by Šupljika *et al*. [1] brings us a greater understanding of the interlinked nature of stress adaptation mechanisms. The complex environmental effects brought by multifactoral stress environments impact the stress responses observed within the simple environments where isolated stresses are employed in routine laboratory work. Nothing makes sense without the light of evolution and an understanding of an organism's natural ecology. It will be fascinating for future studies to identify further links between proteasome assembly and cell wall maintenance, both in *S. cerevisiae*, other fungi and potentially even plants.

## Conflict of interest

The author declares no conflict of interest.

## Author contributions

TDW contributed to the conceptualisation and writing.
